# Adenosquamous Carcinoma of the Stomach: Report of Two Cases

**DOI:** 10.4021/gr2009.01.1261

**Published:** 2009-01-20

**Authors:** Tadashi Terada

**Affiliations:** Department of Pathology, Shizuoka City Shimizu Hospital, Miyakami 1231 Shimizu-Ku, Shizuoka 424-8636, Japan. Email: piyo0111jp@yahoo.co.jp

**Keywords:** Stomach, Adenosquamous carcinoma, Histopathology

## Abstract

The author reports two cases of adenosquamous carcinoma of stomach. The first case is an 87-year-old woman who was admitted to our hospital because of nausea and vomiting. Endoscopy revealed a large type 4 tumor in the stomach, and biopsy showed squamous cell carcinoma. Total gastrectomy, cholecystectomy, splenectomy and lymph node dissection were performed. Pathologically, the gastric tumor consisted of a mixture of adenocarcinoma (30% in area) and squamous cell carcinoma (70% in areas). The adenocarcinoma consisted of signet ring cell carcinoma, poorly differentiated carcinoma, and tubular adenocarcinoma. Carcinoma cells invaded into the serosa. The gall bladder, lymph nodes and peritoneum showed metastases of adenocarcinoma. The patient died of five months after operation. The second case is a granulocyte-colony stimulating factor producing carcinoma. A 77-year-old woman was admitted to our hospital because of epigastralgia. Marked leukocytosis was present without inflammation. Endoscopic examination revealed a large type 3 tumor, and biopsy showed squamous cell carcinoma. Gastrectomy and lymph node dissection was performed. Pathologically the gastric tumor was composed of a mixture of adenocarcinoma (10%) and squamous cell carcinoma (90%). The carcinoma invaded into subserosa. Lymphovascular permeation is present. The adenocarcinoma element consisted of signet ring cell carcinoma. Tumor cells were immunohistochemically positive for granulocyte-colony stimulating factor. The lymph nodes showed metastases of signet ring cell carcinoma. The patient showed systemic metastasis, and died eight months after the operation.

## Introduction

Adenosquamous carcinoma of the stomach is very rare, the incidence being less than 0.5% of all stomach malignancies [1]. This disease has been sporadically reported as case reports [1-4], and comprehensive studies using large series are a few [5-7]. The author reports two cases of adenosquamous carcinoma of the stomach.

## Case report

### Case 1

**Figure 1:**
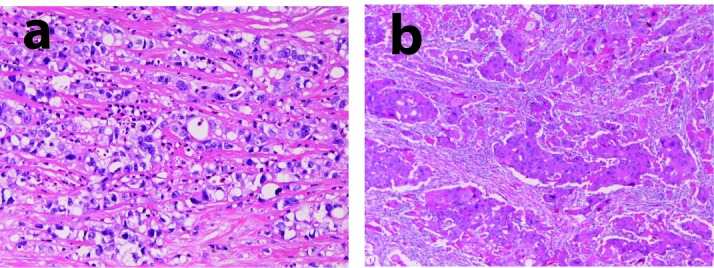
Histology of case 1. Tumor cells are composed of adenocarcinoma (poorly differentiated adenocarcinoma and signet ring cell carcinoma) element (a) and squamous cell carcinoma element (b). HE, x 200.

An 87-year-old woman was admitted to our hospital because of nausea and vomiting. Endoscopy revealed a large type 4 (linitis plastica type) tumor in the stomach, and biopsy showed squamous cell carcinoma. Total gastrectomy, cholecystectomy, splenectomy and lymph node dissection were performed. Grossly, the stomach showed a large type 4 tumor measuring 10 x 8 x 7 cm. Histologically, the gastric tumor consisted of a mixture of adenocarcinoma (30% in area) (Fig. 1a) and squamos cell carcinoma (Fig. 1b) (70% in areas). The adenocarcinoma consisted of signet ring cell carcinoma, poorly differentiated carcinoma, and tubular adenocarcinoma. There was a gradual transition between adenocarcinoma and squamous cell carcinoma. Severe lymphovascular tumor cells permeation was recognized. The carcinoma cells invaded into the serosa. The gall bladder, lymph nodes and peritoneum showed metastases of signet ring cell adenocarcinoma. The patient’s condition deteriorated, and systemic metastasis emerged. The patient died of five months after operation.

### Case 2

**Figure 2:**
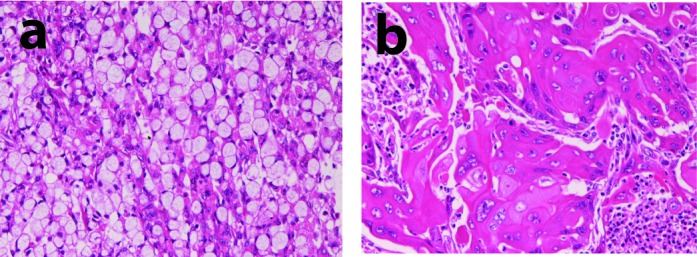
Histology of case 2. Tumor cells are composed of adenocarcinoma (signet ring cell carcinoma) element (a) and squamous cell carcinoma element (b). HE, x 200.

A 77-year-old woman was admitted to our hospital because of epigastralgia. Marked leukocytosis was present without inflammation. Endoscopic examination revealed a large type 3 tumor (ulcerative infiltrating tumor), and biopsy showed squamous cell carcinoma. Gastrectomy and lymph node dissection was performed. Pathologically, the gastric tumor measuring 6 x 5 x 7 cm, composed of a mixture of adenocarcinoma (10%) (Fig. 2a) and squamous cell carcinoma (90%) (Fig. 2b). The carcinoma invaded into subserosa. Lymphovascular permeation was present. The adenocarcinoma element consisted of signet ring cell carcinoma. Immunohistochemical study was performed using Dako Envision methods, as previously reported [8, 9]. Tumor cells were immunohistochemically positive for granulocyte-colony stimulating factor. The lymph nodes showed metastases of signet ring cell carcinoma. The patient showed systemic metastasis, and died eight months after the operation.

## Discussion

The present two tumors consisted of a mixture of adenocarcinoma and squamous cell carcinoma, and gradual transition between the two was present. Thus, the present two tumors were not collision tumors but true adenosquamous carcinomas. The biopsies of the two cases showed squamous cell carcinoma. This is because squamous cell carcinoma predominated over adenocarcinoma in area. Thus, biopsy diagnosis of squamous cell carcinoma of the stomach does not exclude adenosquamous carcinoma. Pure squamous cell carcinoma of the stomach is extremely rare.Clinically, the both patients of the present study showed a rapid clinical course and prognosis was very poor. Studies of large series also reported poor prognosis of adenosquamous carcinoma of the stomach [5, 6].

It is interesting that the present two cases showed metastases of adenocarcinoma but not squamous cell carcinoma. Thus, it seems that the biologic behaviors may be determined by adenocarcinoma element in adenosquamous carcinoma of the stomach. It is also interesting that the present case 2 was granulocyte-colony stimulating factor producing adenosquamous carcinoma. Similar case was reported only once [2]. Much more studies of such cases are required. The origin of adenosquamous carcinoma is unclear. Mori et al [4, 7] considered that adenosquamous carcinoma is derived from squamous transdifferentiation of adenocarcinoma cells, while Mingzzini et al [3] considered that adenosquamous carcinoma of the stomach are derived from totipotential undifferentiated cell of the stomach. The cellular origin of the present cases is unclear. More studies using modern techniques are required.

## References

[1] Toyota N, Minagi S, Takeuchi T, Sadamitsu N (1996). Adenosquamous carcinoma of the stomach associated with separate early gastric cancer (type IIc). J Gastroenterol.

[2] Endo K, Kohnoe S, Okamura T, Haraguchi M, Adachi E, Toh Y, Baba H (2005). Gastric adenosquamous carcinoma producing granulocyte-colony stimulating factor. Gastric Cancer.

[3] Mingazzini PL, Barsotti P, Malchiodi Albedi F (1983). Adenosquamous carcinoma of the stomach: histological, histochemical and ultrastructural observations. Histopathology.

[4] Mori M, Fukuda T, Enjoji M (1987). Adenosquamous carcinoma of the stomach. Histogenetic and ultrastructural studies. Gastroenterology.

[5] Aoki Y, Tabuse K, Wada M, Katsumi M, Uda H (1978). Primary adenosquamous carcinoma of the stomach: experience of 11 cases and its clinical analysis. Gastroenterol Jpn.

[6] Mori M, Iwashita A, Enjoji M (1986). Adenosquamous carcinoma of the stomach. A clinicopathologic analysis of 28 cases. Cancer.

[7] Lee WA, Woo DK, Kim YI, Kim WH (1999). p53, p16 and RB expression in adenosquamous and squamous cell carcinomas of the stomach. Pathol Res Pract.

[8] Terada T, Kawaguchi M (2005). Primary clear cell adenocarcinoma of the peritoneum. Tohoku J Exp Med.

[9] Terada T, Kawaguchi M, Furukawa K, Sekido Y, Osamura Y (2002). Minute mixed ductal-endocrine carcinoma of the pancreas with predominant intraductal growth. Pathol Int.

